# The PKC-β selective inhibitor, Enzastaurin, impairs memory in middle-aged rats

**DOI:** 10.1371/journal.pone.0198256

**Published:** 2018-06-05

**Authors:** Mari N. Willeman, Sarah E. Mennenga, Ashley L. Siniard, Jason J. Corneveaux, Matt De Both, Lauren T. Hewitt, Candy W. S. Tsang, Jason Caselli, B. Blair Braden, Heather A. Bimonte-Nelson, Matthew J. Huentelman

**Affiliations:** 1 Translational Genomics Research Institute, Neurogenomics Division, Phoenix, AZ, United States of America; 2 Arizona Alzheimer’s Consortium, Phoenix, AZ, United States of America; 3 University of Arizona, Evelyn F. McKnight Brain Institute, Tucson, AZ, United States of America; 4 Arizona State University, Department of Psychology, Tempe, AZ, United States of America; Technion Israel Institute of Technology, ISRAEL

## Abstract

Enzastaurin is a Protein Kinase C-β selective inhibitor that was developed to treat cancers. Protein Kinase C-β is an important enzyme for a variety of neuronal functions; in particular, previous rodent studies have reported deficits in spatial and fear-conditioned learning and memory with lower levels of Protein Kinase C-β. Due to Enzastaurin’s mechanism of action, the present study investigated the consequences of Enzastaurin exposure on learning and memory in 12-month-old Fischer-344 male rats. Rats were treated daily with subcutaneous injections of either vehicle or Enzastaurin, and behaviorally tested using the spatial reference memory Morris Water Maze. Rats treated with Enzastaurin exhibited decreased overnight retention and poorer performance on the latter testing day, indicating a mild, but significant, memory impairment. There were no differences during the probe trial indicating that all animals were able to spatially localize the platform to the proper quadrant by the end of testing. RNA isolated from the hippocampus was analyzed using Next Generation Sequencing (Illumina). No statistically significant transcriptional differences were noted. Our findings suggest that acute Enzastaurin treatment can impair hippocampal-based learning and memory performance, with no effects on transcription in the hippocampus. We propose that care should be taken in future clinical trials that utilize Protein Kinase C-ß inhibitors, to monitor for possible cognitive effects, future research should examine if these effects are fully reversible.

## Introduction

Enzastaurin (ENZ) is a Protein Kinase Cβ (PKCβ)-selective inhibitor (Graff 2005) that has undergone human clinical trials as a treatment for various types of difficult to treat chemotherapy-resistant tumors (Ghobrial 2012, Nwankwo 2013). While ENZ treatment was found not to be efficacious in this context [1], it is important to determine the effects of PKC-ß inhibitors, due to the fact that activation of PKC is known to contribute to tumor cell survival, tumor cell proliferation, and decreased patient survival; therefore, this pathway will continue to be an avenue of research for cancer treatment.

PKC-β has shown relationships with learning and memory. PKC-β knock-out mice have been tested on a fear-conditioning paradigm, and were found to have significant deficits in fear conditioning, both in cued and contextual tests. These results were limited to long-term testing, as freezing responses were robust in short-term memory testing [2]. Decreased levels of endogenous PKC-β have been associated with impaired spatial learning and memory in young rats; Colombo (1997) [3] reported that hippocampal PKC-β was correlated with spatial memory, with a higher level of PKC-β in young rats related to better spatial memory. In this report, aged rats exhibited impairment in spatial learning and memory relative to young rats; however, there were no differences in hippocampal PKC-β levels between young and aged rats. Others have shown that aged mice had a 3-fold decrease in both hypothalamic and cortical PKC-β [4], along with other misregulations of proteins involved in neuronal signaling and in age-related phenotypes and diseases.

It is biologically feasible that PKC-β could impact cognition. Specifically, PKC-β may lead to memory impairments via direct effects on long-term potentiation (LTP) mechanisms. PKC-ß has been associated with LTP through phosphorylation of neurogranin, which is thought to play a role in calcium homeostasis, a process that is crucial for LTP [5], and neuromodulin, which is involved in neurite growth and neurotransmitter release in the hippocampus [6]. PKC-ß’s effects on cognition may also be related to long-term depression (LTD) and PKC-ß’s effects on the apoE and/or cholinergic pathways. Calcium homeostasis is deregulated with aging [7], and activation of the amyloidogenic pathway alters calcium signaling pathways involved in cognition, resulting in increased intracellular calcium levels that are conducive to long-term depression (LTD) mechanisms, which have the potential to mitigate LTP and learning [8]. Lower PKC-β activity in the cerebral cortex is also associated with deficiency in apolipoprotein E (apoE) [9]. ApoE deficiency has been shown to decrease cholinergic levels and impair memory [10], and ApoE-deficient mice often serve as a model for cholinergic impairment-induced memory deficits.

While these findings implicate PKC-β as a critical enzyme in learning and memory function, few studies [11] have tested the effect of PKC-β inhibitors on learning or memory tasks. In this study we evaluated a PKC-β inhibitor previously utilized in the human clinic to explore the effects of direct PKC-β inhibition on spatial learning and memory in middle-aged rats. We also evaluated RNA expression after behavioral testing to assess transcriptional changes in the hippocampus, an area intimately involved with spatial learning and memory.

## Methods

### Subjects

Middle-aged (12 months) virgin male Fischer-344 rats born and raised at the National Institute on Aging colony at Harlan Laboratories (Indianapolis, IN) were used. The rats were bred for research purposes, and were pair housed at Arizona State University, had exposure to food and water ad-lib, and were maintained on a 12-h light/dark cycle at 23°C. Experimental procedures were approved by the Arizona State University Institutional Animal Care and Use Committee and adhered to Guidelines for the Care and Use of Laboratory Animals and NIH standards.

### Treatments

Rats were randomly divided into two treatment groups, receiving either vehicle (n = 10) or ENZ (n = 8). Rats received either daily subcutaneous injections of ENZ (25mg/kg; 0.4mL) (Selleck Chemicals, Houston, TX) suspended in polyethylene glycol (PEG, Sigma-Aldrich, St. Louis, MO) or vehicle (100% PEG) for three days concurrent to behavioral testing. Injections started the first day of behavioral testing. Maze testing commenced 30–45 minutes after injection. Animals were sacrificed immediately after the probe trial on day 3 of testing.

### Apparatus

Spatial reference memory was tested using the Morris maze (MM) task [12,13]. The maze consisted of a round tub (188 cm in diameter) filled with room temperature water tinted with black non-toxic paint. Rats were placed in the maze from one of four locations (North, South, East, or West) and had 60 sec to locate the platform, which remained in a fixed location in the Northeast (NE) quadrant. After 15 sec on the platform, the rat was placed into a heated cage until the next trial. Animals were tested in two squads, with nine rats in each squad, counterbalanced for drug treatment. The first trial was completed for each rat in the group, then the second, etc., as done previously [14,15]. Rats received six trials/day for three days, with a 15 min delay given between trials three and four [16]. A video camera recorded each rat and a tracking system (Ethovision XT 5.1, Noldus) analyzed each rat’s path. The dependent variable was swim distance (cm). Unless otherwise noted, two-tailed tests were used, and alpha was set at 0.05. Data were analyzed with a repeated measures ANOVA with Treatment as the between variable and Days (days 1–3), and Trials (trials 1–6) as the within variables.

Since we have shown that some treatments can impact retention of the platform location overnight in older rats [17–19], we assessed the effects of ENZ on overnight retention by comparing swim distance from the last test trial on the first day (trial six on day one) to the first test trial the next day (trial one on day two), and for the overnight interval from day two to three (from trial six on day two to trial one on day three).

To assess platform localization, a probe trial was given on trial seven of the last day of testing, whereby the platform was removed from the maze. For the probe, percent of total swim distance (cm) traveled in the target NE quadrant (i.e., quadrant that contained the platform) as compared to the opposite Southwest (SW) quadrant was the dependent measure [14,20]. Additional dependent variables included the frequency of crossing into the platform zone, the NE quadrant, and the SW quadrant.

### Next generation mRNA sequencing and analysis

In order to create a genome-wide transcriptional profile of the tissue, RNA sequencing was performed on the hippocampus of the animals. Next Generation RNA Sequencing (RNA-Seq) was used in order to analyze transcriptional differences across the genome of the rats in the study. Immediately after the probe trial on day 3 of testing, animals were anesthetized with isofluorane gas and decapitated using a guillotine, and brains were rapidly dissected and frozen. Dissected tissues were stored in pre-weighed microcentrifuge tubes at -70°C until analysis. Dissection of the CA1/CA2 region of the hippocampus (plates 39–42) was according to plate designations in Paxinos and Watson (1998). The brain was cut in the coronal plane to obtain access to the ventral CA1/CA2 region of the hippocampus. The dentate gyrus and the alveus were excluded. RNA was isolated from the left hippocampal region of each animal using TRIzol® Reagent (Invitrogen) and then purified with the RNeasy Kit (Qiagen) using a modified version of the RNeasy Trizol procedure. Rather than precipitating with 0.5mL isopropanol per 1mL Trizol, 1 volume of 70% ethanol was used. After precipitation, the RNeasy protocol was used. However, rather than using 700ul of RW1, half the volume was used, and DNase was used after this step. After the DNase step, another 350ul of RW1 was used, and the rest of the protocol was followed as originally written.

Concentration of the RNA was determined using Ribogreen (Invitrogen). Quality of RNA was assessed with 2100 Bioanalyzer (Agilent Technologies) and RINs were all above 8.3.1ug of total RNA for each sample was prepared for sequencing using the TruSeq RNA Sample Preparation Kit (Illumina Inc), following the manufacturers protocol. The final sample library was validated on the 2100 Bioanalyzer (Agilent Technologies), quantified using qPCR (7900HT, Applied Biosystems; KAPA Biosystems), and sequenced by 100bp paired-end sequencing on a HiSeq2000 (Illumina Inc.).

An average of 8.1 million reads were dedicated to each sample. Reads were trimmed of adapter sequences (AlienTrimmer, 0.3.2), aligned to the rn5 reference genome (STAR, 2.4.0a) which is the genome of *Rattus norvegicus* sequenced by the Rat Genome Sequencing Consortium, and quantified at the gene level (featureCounts, 1.4.4). Principal components analysis (PCA) identified two samples that were significant outliers and were removed prior to analysis. Differential expression (DESeq2) was conducted at the group level (ENZ treated vs. vehicle treated) using the Wald test. To account for the parallelized assessment of multiple hypotheses, corrected p-values were calculated using the Benjamini-Hochberg false discovery rate procedure.

## Results

### Morris Maze

**Fig 1:**
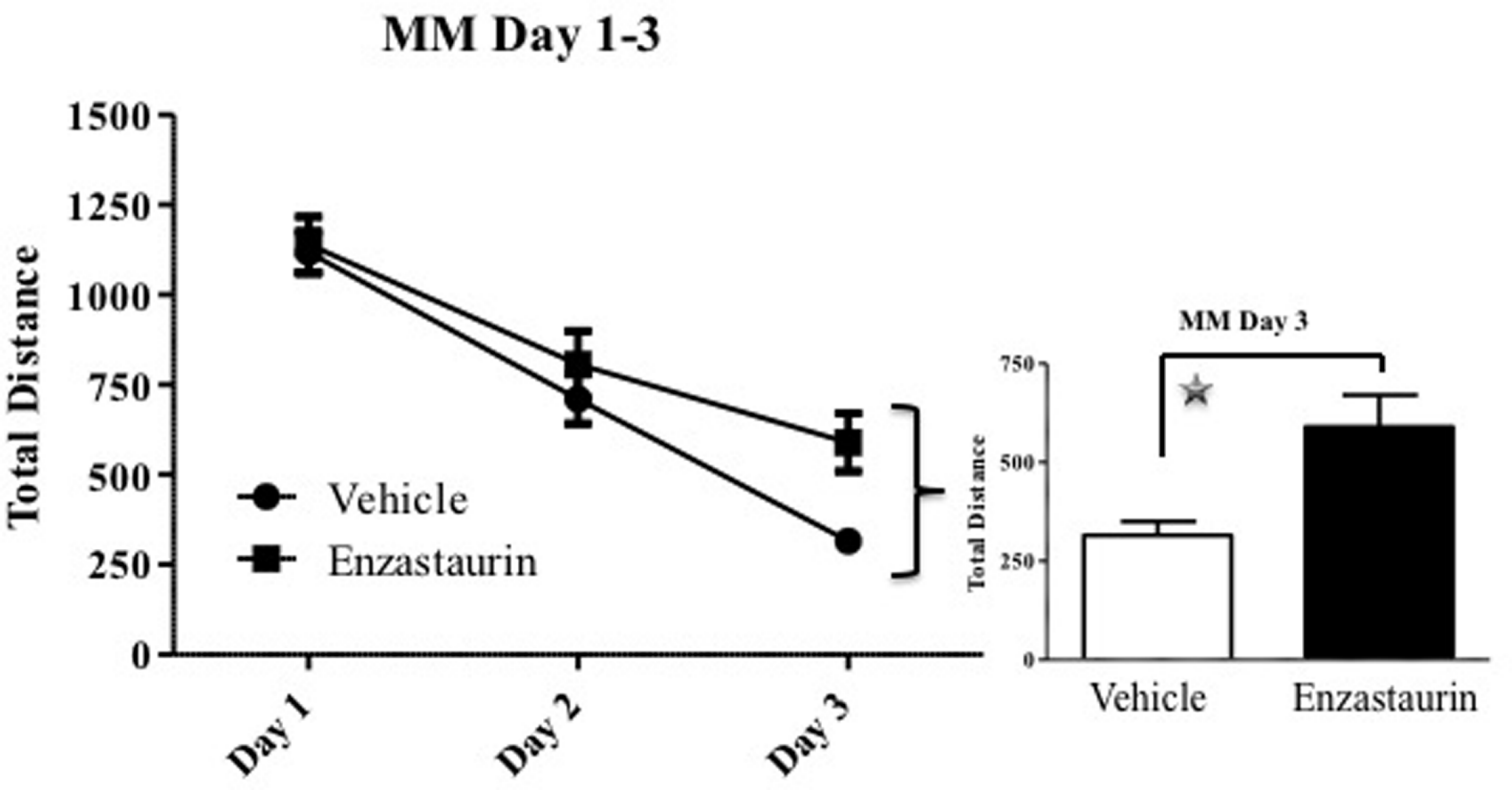
ENZ treatment results in a modest impairment on the Morris Water Maze task of hippocampal-dependent reference memory. ENZ treatment increased the total swim distance only on Day 3 of testing, indicating an impairment in learning and memory. However, there was no significant difference in total swim distance for either Day 1 or 2.

**Fig 2:**
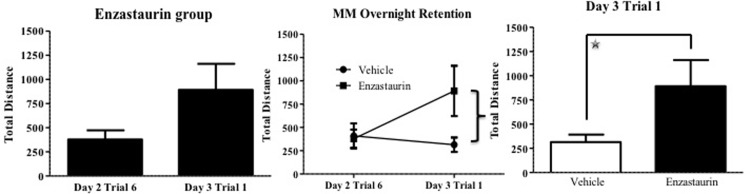
ENZ treatment impairs overnight retention in the Morris Water Maze. ENZ treatment led to increased swim distance on the first trial of the final testing day, in comparison to the final trial on the previous day. This is indicative of an absence of overnight retention. Comparatively, vehicle treatment led to similar performance for the two trials, indicating that overnight retention was maintained.

Fig 1 shows the mean distance to platform scores ± SE for each treatment group across the three days of MM testing, collapsed across trials. There was no main effect of Treatment collapsed across all days and trials (main effect p > 0.10). However, while there was a null Treatment x Day interaction, likely due to the similar performance of the groups on days 1 and 2, there were divergent scores on day 3. In fact, a post hoc ANOVA showed that the ENZ group had a higher mean distance score on day 3, indicating poorer performance on this lattermost testing day [F(1,16) = 4.54; p < 0.05]. As shown in Fig 2, the ENZ group also showed deficits in overnight retention leading to this last day, with an increase in swim distance across the second overnight interval (day two to day three) [F(1,7) = 3.69; p < 0.05, one tailed], while the Vehicle group maintained performance across this overnight interval [F(1,9) = 0.28; p = 0.61]. Further analyses showed that the ENZ group exhibited poorer performance than the Vehicle group on the first trial of day 3, which was the second overnight retention trial [F(1,16) = 5.13; p < 0.05].

**Fig 3:**
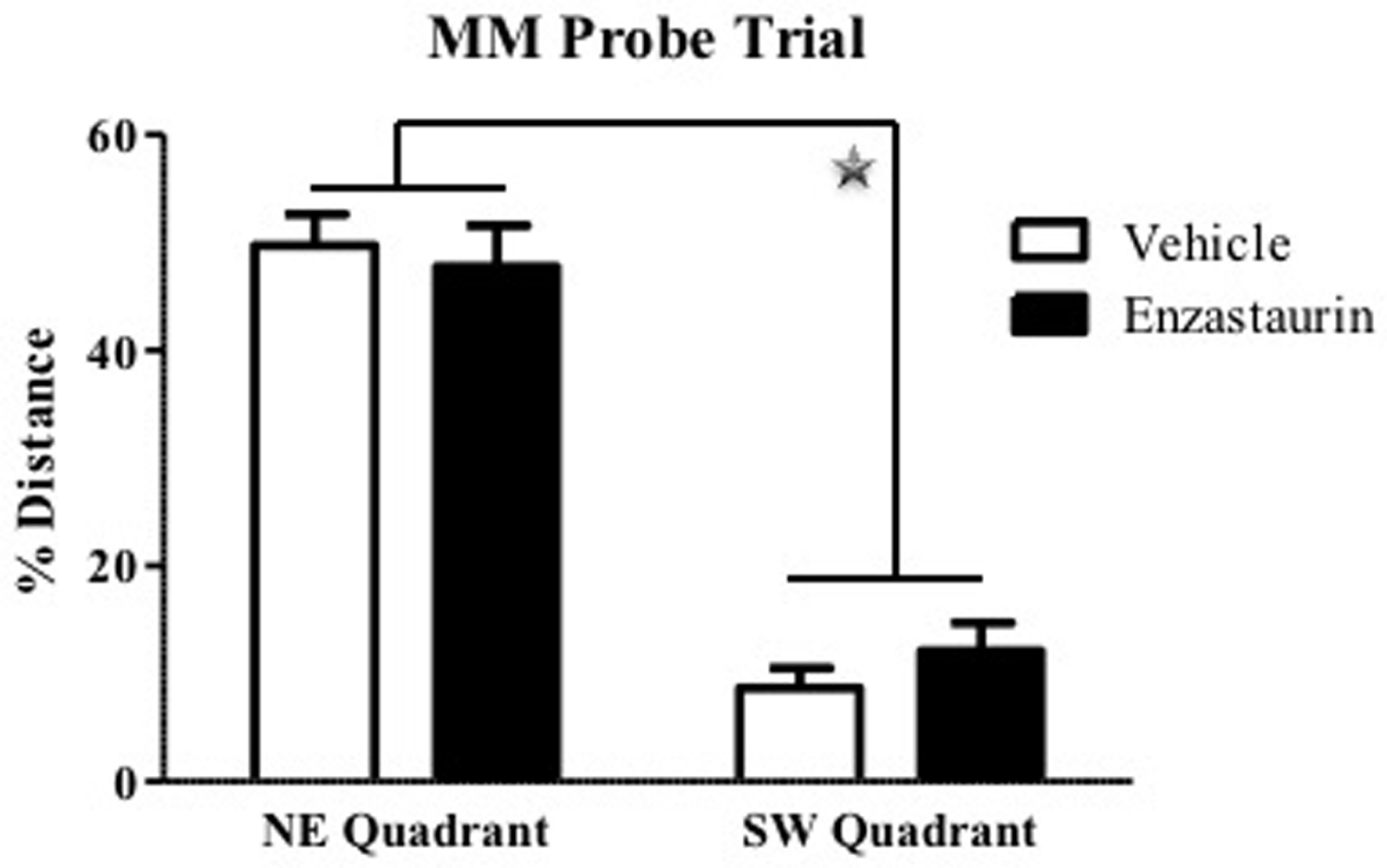
ENZ treated and control groups demonstrate similar performance in the Morris Water Maze probe trial. During the probe trial, both ENZ-treated and vehicle-treated groups swam for a significantly higher percentage of distance in the quadrant that included the platform during previous testing days in comparison to the opposite quadrant. This indicates that ENZ treatment did not decrease the ability to locate the quadrant in which the platform should be.

For the probe trial, there was a Quadrant main effect, with a greater percent swim distance in the NE (target), versus the SW (opposite), quadrant [F(1, 16) = 109.81; p<0.0001] (Fig 3). The absence of a Treatment main effect or Treatment x Quadrant interaction for the probe trial indicated that ENZ did not impact the ability to localize the target quadrant differently than that of the animals receiving vehicle. There were no group differences for platform location crossings on the probe trial.

### Next Generation mRNA sequencing

**Fig 4:**
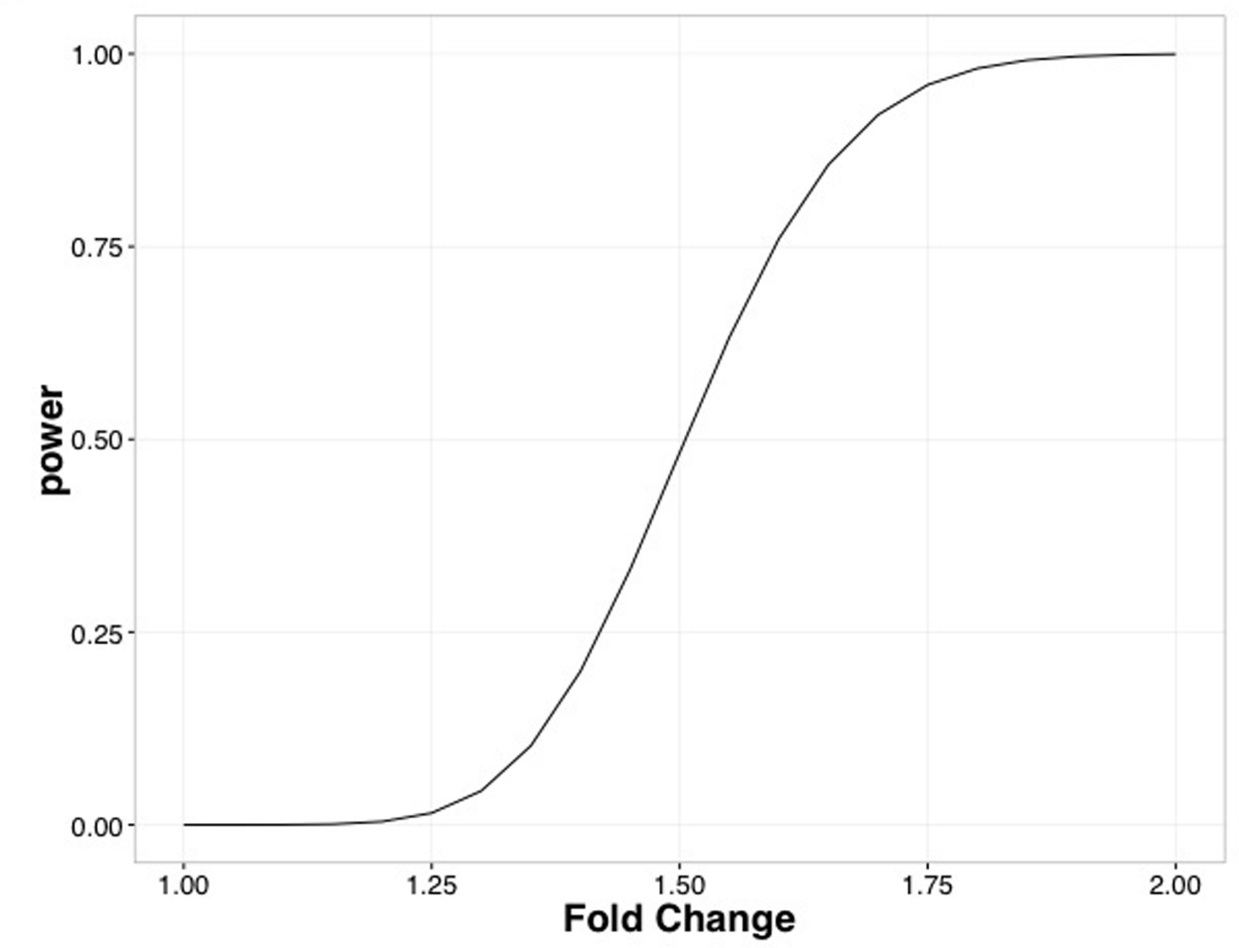
Power analysis. Given our study design and post hoc calculations of variance, this plot shows our power to detect different effect sizes.

RNA sequencing of the hippocampus (CA1/CA2 sub-regions), extracted immediately from animals following the probe trial, showed no significant differences in the expression of transcripts following false discovery rate correction. The top 5 genes based on un-corrected p-value are listed in Table 1. In the current experimental design, given the study size (n = 7 per group), Fig 4 shows that we had the power to detect different effect sizes, the median variance observed across all genes and samples, and the specified false discovery rate (0.05/25000). Coefficient of variation (CV) was calculated from the data by dropping genes with median (counts) < 5, and taking the median CV across all genes. As a proxy for average normalized counts, median normalized counts were found across each gene, and the median of the gene-medians was used. Based on our power calculations we were 80% powered to detect a fold change of approximately 1.6.

**Table 1:** The “top 5” hits.

gene	ensemblID	baseMean	log2FoldChange	lfcSE	stat	pvalue	padj
Jak3	ENSRNOG00000018669	83.30357485	-0.498088696	0.156648305	-3.179662212	0.0014	0.999
Hba2	ENSRNOG00000047321	425.4349624	0.323115949	0.104336314	3.096869509	0.0019	0.999
Chpf2	ENSRNOG00000010466	359.9593506	-0.202950886	0.066339508	-3.059276324	0.0022	0.999
Serping1	ENSRNOG00000007457	32.22528476	-0.65845592	0.215354286	-3.057547315	0.0022	0.999
Mavs	ENSRNOG00000025295	126.3450306	0.282988815	0.094113608	3.006885193	0.0026	0.999

No significant transcriptional changes were found using the gene expression level using a Wald test.

## Discussion

PKCβ’s role in learning and memory, and related brain effects, remain largely unknown. The present study examined the effects of ENZ, a selective PKC-ß inhibitor, on learning and memory, as well as transcriptional effects in the hippocampus in male rats in order to address this concern. To our knowledge, this is the first report evaluating the cognitive consequences of ENZ treatment. Here, we show that daily treatment with ENZ in middle-aged male rats resulted in impaired performance on two measures of spatial reference memory, as evaluated on the MM. Specifically, ENZ-treated animals showed a greater distance traveled at the end of maze testing, on the last day of testing, indicating poorer spatial reference memory. There was also a greater increased distance over the last overnight interval, demonstrating poorer spatial memory retention, compared to Vehicle-treated counterparts. The probe trial, whereby the platform was removed, showed no differences between groups in percent swim distance in the previously platformed quadrant versus the opposite quadrant. Thus, by the end of testing all groups were able to localize the spatial position of the platform within the given trial time. Of note, drug administration in the current study was acute and was initiated only when behavioral testing started. Thus, it is unknown whether inhibition of PKC-β over a long-term period would continue to impair spatial memory retention, and whether effects are reversible with treatment discontinuation. These questions are important in determining whether chronically lower levels of PKC-β in patients lead to poor memory retention, and whether treatment to increase PKC-β levels would improve memory retention. Equally important is whether PKC-β inhibition has an impact on other cognitive domains, such as non-spatial cognition and working memory.

Our findings correspond with work showing that the PKC-βI isozyme inhibitor Go 6976 impairs inhibitory avoidance learning in rats [11]; effects of these compounds on other forms of learning and memory are, as of yet, unknown. Of note, other changes in PKC family levels have also been implicated in altered cognition. Variability in the gene encoding PKCα (PRKCA) was associated with changes in memory capacity [21], and PRKCA genotype is important in determining how episodic memories are retrieved [22]. Huprine X, an anticholinesterastic drug, has been shown to improve learning and memory in aged mice, with an observed increase in PKCα in brain tissue post-sacrifice [23]. Blood brain barrier (BBB) disruption can lead to inflammation and other neuronal changes that could affect learning and memory mechanisms, and inhibition of PKCβ has been considered a therapeutic approach for patients with disturbed BBB [24]. Further, Enzastaurin itself has been shown to decrease BBB leakiness in leptin receptor deficient mice, and return hippocampal function to normal [25]. This provides further evidence that the PKC family plays an important role in learning and memory mechanisms.

For the transcripts analyzed here using the rn5 reference genome, as sequenced by the Rat Genome Sequencing Consortium, there were no significant differences in the transcriptional expression levels between the ENZ-treated and control groups. One interpretation of this null finding is that the consequences of ENZ on memory may be due to non-genomic impacts on learning and memory. This result is in line with the findings of Paratcha et al., (2000) that another PKCβ inhibitor impaired fast (e.g., non-genomic) mechanisms underlying inhibitory avoidance learning. In the present study, we observed modest changes in spatial reference memory after just 2 days of drug administration and behavioral testing. Future studies will be needed to determine whether effects of ENZ persist, and continue with extended chronic treatment. Given that our study exposed rats to ENZ acutely, for only three days, there may not have been enough time for long-term, or genomic, effects to become evident. Liu, et al. (2015) [26] found that with long-term treatment of another selective PKCβ inhibitor, roboxistaurin, Caveolin-3 expression decreased. Long-term treatment with ENZ may also lead to similar changes. Caveolin-3, along with other caveolin isoforms, may regulate neuroinflammatory responses as suggested in a study that found increased lesions after traumatic brain injury in Caveolin knock-out mice. [27]. While our data do not indicate a change in Caveolin-3 expression with short-term ENZ treatment, it is important to determine whether long-term treatment would result in changes. There are many other possible explanations for our lack of observed gene expression changes. We profiled hippocampal-dependent memory and selected the hippocampus for RNA profiling. It is possible that another brain region is involved in the behavior effects we observe and may demonstrate gene expression changes. It also could be possible that the effects of ENZ are cell-type specific and our hippocampal homogenate RNA sequencing approach failed to identify these changes due to a signal to noise complicated by cellular transcriptomes that were largely not altered by ENZ treatment. Lastly, it is possible that the effects on transcription were subtle enough that a larger study size was needed to detect them.

Of note, all of the animals utilized in this study were males, thus the results presented here may not apply to the aging female. Interestingly, Cordey et al. (2003) [28] reported that estradiol activates PKC in several cell types, and is neuroprotective against amyloid-β toxicity in vitro. How the many types of female aging trajectories, including variations in reproductive senescence and subsequent hormone therapies, impact PKC function and cognition throughout aging remains largely unknown.

Finally, it is important to note that we utilized a single dosing strategy in this study, aiming to use a low efficacious dose to be as selective as possible (e.g. to avoid potential off target effects of higher drug concentrations). The dose used in the current study was much lower than many prior publications testing ENZ; doses in the range of 30mg/kg to 100mg/kg have been used before in mouse models, and in some instances these doses were administered two or three times daily [29–32]. The ENZ dose selected in the current study was based on previous publications whereby an efficacious physiological effect was observed at a comparably low dosage (1.25mg per day or ~25mg/kg in a mouse [24]; 2 doses of 15mg/kg per day [33], 50mg/kg per day [25]). A different dosing approach may yield different cognitive and transcriptional effects and relationships.

The current study testing ENZ, a PKC-β inhibitor, supports accumulating evidence that the PKC-β pathway can regulate learning and memory. Moreover, the presented data are important because they indicate drugs that inhibit PKCβ may negatively impact brain functioning. More investigation into how the PKC family is involved in the neural processes of learning and memory is needed, especially as many of the same mechanisms involved in tumor growth may be necessary for neuron growth and adaptation, such as LTP and LTD. The behavioral breadth and permanence of the mnemonic effects, as well as the underlying brain mechanisms, are important next steps to consider as systematic scientific evaluations.
